# Poly[μ_6_-adipato-diaquadi-μ_2_-oxalato-digadolinium(III)]

**DOI:** 10.1107/S1600536810036111

**Published:** 2010-09-15

**Authors:** Zhi-Feng Li, Chun-Xiang Wang

**Affiliations:** a School of Materials & Chemical Engineering, Jiangxi University of Science and Technology, Ganzhou 341000, People’s Republic of China

## Abstract

In the centrosymmetric title compound, [Gd_2_(C_6_H_8_O_4_)(C_2_O_4_)_2_(H_2_O)_2_]_*n*_, the Gd^3+^ cations are each coordinated by nine O atoms, three from adipate anions, two from oxalate anions and one from an aqua ligand, completing a distorted tricapped trigonal-prismatic geometry. These tricapped trigonal prisms are bridged by the adipate ligands, generating layers lying parallel to (010). The coordination polymer layers are linked into a three-dimensional framework by the rigid oxalate ligands. The adipate and oxalate ions are all located on centers of inversion. A part of the adipate anion is disordered over two positions in a 0.75:0.25 ratio.

## Related literature

For structures involving adipate ligands and lanthanide ions, see: Dimos *et al.* (2002 ▶). For structures involving oxalate ligands and lanthanide ions, see: Trombe & Mohanu (2004 ▶).
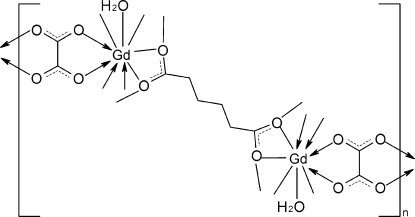


## Experimental

### 

#### Crystal data

[Gd_2_(C_6_H_8_O_4_)(C_2_O_4_)_2_(H_2_O)_2_]
*M* *_r_* = 670.70
Triclinic, 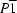

*a* = 6.815 (4) Å
*b* = 6.982 (4) Å
*c* = 8.997 (7) Å
α = 104.759 (10)°
β = 108.11 (1)°
γ = 104.320 (7)°
*V* = 367.8 (4) Å^3^
*Z* = 1
Mo *K*α radiation
μ = 9.02 mm^−1^
*T* = 295 K
0.23 × 0.11 × 0.08 mm

#### Data collection

Bruker SMART APEXII CCD area-detector diffractometer
Absorption correction: multi-scan (*SADABS*; Sheldrick, 2003 ▶) *T*_min_ = 0.241, *T*_max_ = 0.491
1751 measured reflections
1236 independent reflections
1157 reflections with *I* > 2σ(*I*)
*R*_int_ = 0.013

#### Refinement

*R*[*F* ^2^ > 2σ(*F* ^2^)] = 0.023
*wR*(*F* ^2^) = 0.059
*S* = 1.05
1236 reflections
127 parameters
2 restraints
H atoms treated by a mixture of independent and constrained refinement
Δρ_max_ = 1.56 e Å^−3^
Δρ_min_ = −1.41 e Å^−3^

### 

Data collection: *APEX2* (Bruker, 2004 ▶); cell refinement: *SAINT* (Bruker, 2004 ▶); data reduction: *SAINT*; program(s) used to solve structure: *SHELXS97* (Sheldrick, 2008 ▶); program(s) used to refine structure: *SHELXL97* (Sheldrick, 2008 ▶); molecular graphics: *SHELXTL* (Sheldrick, 2008 ▶); software used to prepare material for publication: *SHELXTL*.

## Supplementary Material

Crystal structure: contains datablocks I, global. DOI: 10.1107/S1600536810036111/su2209sup1.cif

Structure factors: contains datablocks I. DOI: 10.1107/S1600536810036111/su2209Isup2.hkl

Additional supplementary materials:  crystallographic information; 3D view; checkCIF report

## Figures and Tables

**Table 1:** Hydrogen-bond geometry (Å, °)

*D*—H⋯*A*	*D*—H	H⋯*A*	*D*⋯*A*	*D*—H⋯*A*
O7—H7*A*⋯O3^i^	0.84 (6)	2.00 (5)	2.799 (4)	160
O7—H7*B*⋯O6^ii^	0.83 (7)	2.07 (7)	2.888 (5)	168

Symmetry codes: (i) 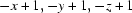; (ii) 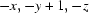.

**Fig. 1.**
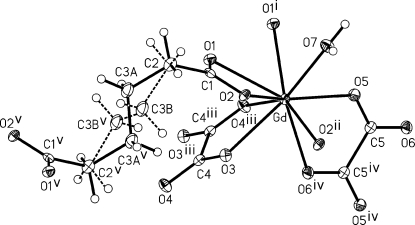
The fragment of the molecuar structure of the title compound, with the atom-numbering scheme. Displacement ellipsoidsare drawn at the 30% probability level and H atoms are shown as small spheres of arbitrary radii. Symmetry codes: (i) -x, 1 - y, 1 - z; (ii) 1 - x, 1 - y, 1 - z; (iii) -x, - y, 1- z; (iv) - x, - y, -z; (v) 1 - x, 1 - y, 2 - z.

**Fig. 2.**
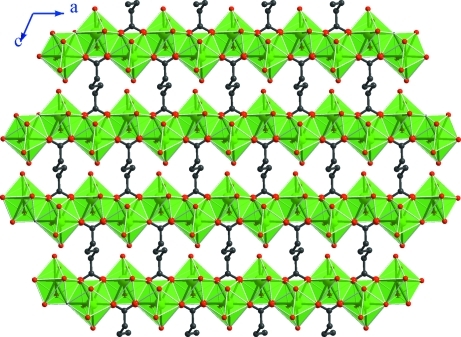
A view along the b-axis of the two-dimensional layer structure formed by the connectivity between the Gd atoms and the adipate moieties present in the title compound [the C-bound H-atoms and atom C3B have been omitted for clarity].

**Fig. 3.**
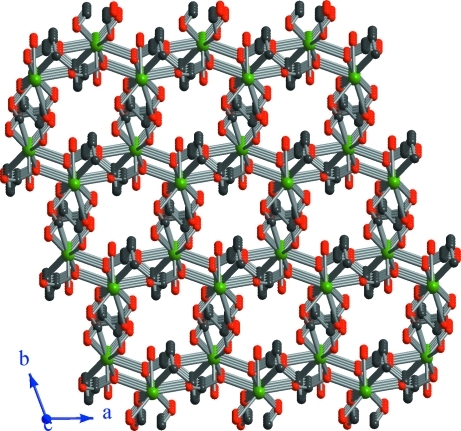
A view along the c-axis of the three-dimensional framework of the title compound [the C-bound H-atoms and atom C3B have been omitted for clarity].

## supplementary crystallographic information

### Comment

As shown in Fig. 1 the asymmetric unit of the title compound consists of one Gd^3+^ cation,  half of an adipate anion, two half oxalate anions and one aqua ligand. The Gd atom is coordinated by nine oxygen atoms, in which four oxygen atoms are from three adipate anions, four from two oxalate anions and one from a water molecule, to form a SmO_9_ polyhedron with a distorted tricapped trigonal-prismatic geometry. The Gd—O(adipate) distances vary in the range of 2.430 (4)–2.575 (4) Å (average 2.512 (4) Å), which is nearly identical to the value of 2.425 (8)–2.611 (8) Å (average 2.471 (8) Å) observed in Gd_2_(C_6_H_8_O_4_)_3_(H_2_O)_4_ (Dimos *et al.*, 2002). The Gd—O(oxalate) distances are in the range of 2.385 (4)–2.540 (4) Å, which are usual for lanthanide oxalates complexes (Trombe & Mohanu, 2004). In the title complex, the adipate anions are located on inversion centers and atom C3 is positionally disordered (C3A and C3B; occupancies 0.75/0.25). Two carboxylate oxygen atoms chelate one Gd atom with each oxygen atom additionally bonded to another Gd atom. To the best of our knowledge, this η^2^,µ_3_-η^2^,µ_3_-chelating-bridging octadentate coordination mode of the adipate ligand has not been reported previously.

Through the terminal carboxylato bridging interactions, the GdO_9_ polyhedra are edged-shared to generate metal-oxygen chains extending infinitely along [100], in which the adjacent Gd···Gd distances are 4.23 (3) Å and 4.25 (2) Å, respectively. Along  [001] the chains are linked by the adipate anions into layers parallel to (010) (Fig. 2). Two symmetry independent oxalate ions are also located on centers of inversion and act as double bidentate (tetradentate) ligands in a linear chain, which connect Gd atoms to form zigzag chains along [001]. Through the oxalate and adipate ligand bridging interactions, the Gd atoms build up a three-dimensional open framework with the channels propagating in [001] (Fig. 3). The aqua ligand provides H-bond donors which participate in O-H···O hydrogen bonds with the oxalate atoms O3 and O6 (Table 1).

### Experimental

A mixture of GdCl_3_.6H_2_O(1.00 mmol, 0.36 g), oxalic acid (0.50 mmol, 0.05 g), adipic acid (0.50 mmol, 0.07 g), NaOH (2.00 mmol, 0.08 g) and H_2_O (10.0 ml) was heated in a 23 ml stainless steel reactor with a Teflon liner at 443 K for 48 h. On cooling a small amount of colorless plate-like crystals were obtained.They were filtered off and washed with water and acetone.

### Refinement

Atom C3 of the adipate anion is positionally disordered (C3A and C3B) and these atoms were refined with occupancies of 0.75/0.25. The water H-atoms were located in difference Fourier maps and were refined with distance restraints: O—H distance of 0.84 (2) Å) and *U*_iso_(H) = 1.5*U*_eq_(O). The C-bound atoms were included in calculated positions and treated as riding atoms: C–H = 0.97 Å and *U*_iso_(H) = 1.2*U*_eq_(C). The highest density peak and deepest hole are located at 0.97 Å and 0.89 Å, respectively, from the Gd atom.

### Crystal data

**Table:**

[Gd_2_(C_6_H_8_O_4_)(C_2_O_4_)_2_(H_2_O)_2_]	*Z* = 1
*M**_r_* = 670.70	*F*(000) = 312
Triclinic, *P*1	*D*_x_ = 3.028 Mg m^−^^3^
Hall symbol: -P 1	Mo *K*α radiation, λ = 0.71073 Å
*a* = 6.815 (4) Å	Cell parameters from 238 reflections
*b* = 6.982 (4) Å	θ = 2.6–28.3°
*c* = 8.997 (7) Å	µ = 9.02 mm^−^^1^
α = 104.759 (10)°	*T* = 295 K
β = 108.11 (1)°	Plate, colorless
γ = 104.320 (7)°	0.23 × 0.11 × 0.08 mm
*V* = 367.8 (4) Å^3^	

### Data collection

**Table:**

Bruker SMART APEXII CCD area-detector diffractometer	1236 independent reflections
Radiation source: fine-focus sealed tube	1157 reflections with *I* > 2σ(*I*)
graphite	*R*_int_ = 0.013
φ and ω scans	θ_max_ = 25.0°, θ_min_ = 2.6°
Absorption correction: multi-scan (*SADABS*; Sheldrick, 2003)	*h* = −7→8
*T*_min_ = 0.241, *T*_max_ = 0.491	*k* = −7→8
1751 measured reflections	*l* = −9→10

### Refinement

**Table:**

Refinement on *F*^2^	Primary atom site location: structure-invariant direct methods
Least-squares matrix: full	Secondary atom site location: difference Fourier map
*R*[*F*^2^ > 2σ(*F*^2^)] = 0.023	Hydrogen site location: inferred from neighbouring sites
*wR*(*F*^2^) = 0.059	H atoms treated by a mixture of independent and constrained refinement
*S* = 1.05	*w* = 1/[σ^2^(*F*_o_^2^) + (0.0451*P*)^2^] where *P* = (*F*_o_^2^ + 2*F*_c_^2^)/3
1236 reflections	(Δ/σ)_max_ < 0.001
127 parameters	Δρ_max_ = 1.56 e Å^−^^3^
2 restraints	Δρ_min_ = −1.41 e Å^−^^3^

### Special details

**Table:**

Geometry. All e.s.d.'s (except the e.s.d. in the dihedral angle between two l.s. planes) are estimated using the full covariance matrix. The cell e.s.d.'s are taken into account individually in the estimation of e.s.d.'s in distances, angles and torsion angles; correlations between e.s.d.'s in cell parameters are only used when they are defined by crystal symmetry. An approximate (isotropic) treatment of cell e.s.d.'s is used for estimating e.s.d.'s involving l.s. planes.
Refinement. Refinement of *F*^2^ against ALL reflections. The weighted *R*-factor *wR* and goodness of fit *S* are based on *F*^2^, conventional *R*-factors *R* are based on *F*, with *F* set to zero for negative *F*^2^. The threshold expression of *F*^2^ > σ(*F*^2^) is used only for calculating *R*-factors(gt) *etc*. and is not relevant to the choice of reflections for refinement. *R*-factors based on *F*^2^ are statistically about twice as large as those based on *F*, and *R*- factors based on ALL data will be even larger.

### Fractional atomic coordinates and isotropic or equivalent isotropic displacement parameters (Å^2^)

**Table:**

	*x*	*y*	*z*	*U*_iso_*/*U*_eq_	Occ. (<1)
Gd	0.15528 (4)	0.34583 (3)	0.36187 (3)	0.01206 (12)	
O1	0.1523 (6)	0.5226 (6)	0.6494 (4)	0.0175 (8)	
O2	0.4788 (6)	0.5964 (5)	0.6393 (4)	0.0156 (8)	
O3	0.2680 (6)	0.1539 (6)	0.5394 (4)	0.0168 (8)	
O4	0.1538 (6)	−0.0817 (6)	0.6500 (5)	0.0191 (8)	
O5	−0.0130 (7)	0.2517 (6)	0.0669 (4)	0.0191 (8)	
O6	−0.1324 (7)	0.0169 (6)	−0.1924 (5)	0.0210 (9)	
O7	0.2703 (7)	0.6881 (6)	0.3356 (5)	0.0218 (9)	
H7A	0.403 (5)	0.763 (10)	0.370 (8)	0.033*	
H7B	0.218 (12)	0.757 (10)	0.281 (8)	0.033*	
C1	0.3614 (9)	0.6107 (8)	0.7255 (7)	0.0144 (11)	
C2	0.4651 (10)	0.7225 (9)	0.9107 (7)	0.0193 (12)	
H2A	0.4012	0.8285	0.9405	0.023*	0.75
H2B	0.6218	0.7953	0.9448	0.023*	0.75
H2C	0.3517	0.7421	0.9503	0.023*	0.25
H2D	0.5696	0.8616	0.9373	0.023*	0.25
C3A	0.4343 (15)	0.5720 (14)	1.0070 (10)	0.0262 (17)	0.75
H3A1	0.4800	0.6550	1.1242	0.031*	0.75
H3A2	0.2790	0.4864	0.9633	0.031*	0.75
C3B	0.588 (4)	0.598 (4)	1.005 (3)	0.0262 (17)	0.25
H3B1	0.6830	0.5544	0.9531	0.031*	0.25
H3B2	0.6791	0.6879	1.1213	0.031*	0.25
C4	0.1222 (8)	0.0203 (8)	0.5547 (6)	0.0140 (11)	
C5	−0.0433 (9)	0.0774 (8)	−0.0367 (7)	0.0143 (11)	

### Atomic displacement parameters (Å^2^)

**Table:**

	*U*^11^	*U*^22^	*U*^33^	*U*^12^	*U*^13^	*U*^23^
Gd	0.00971 (18)	0.01274 (17)	0.00989 (17)	0.00183 (12)	0.00225 (12)	0.00217 (11)
O1	0.016 (2)	0.0173 (19)	0.0148 (19)	0.0059 (16)	0.0033 (16)	0.0017 (16)
O2	0.013 (2)	0.0160 (19)	0.0132 (19)	0.0034 (16)	0.0050 (16)	0.0007 (15)
O3	0.011 (2)	0.0182 (19)	0.0161 (19)	0.0011 (16)	0.0029 (16)	0.0050 (16)
O4	0.013 (2)	0.021 (2)	0.020 (2)	0.0031 (17)	0.0015 (16)	0.0105 (17)
O5	0.021 (2)	0.018 (2)	0.0131 (19)	0.0066 (17)	0.0035 (16)	0.0023 (16)
O6	0.027 (2)	0.021 (2)	0.014 (2)	0.0086 (18)	0.0061 (18)	0.0053 (17)
O7	0.017 (2)	0.020 (2)	0.029 (2)	0.0045 (18)	0.0081 (19)	0.0134 (18)
C1	0.016 (3)	0.011 (2)	0.013 (3)	0.006 (2)	0.002 (2)	0.005 (2)
C2	0.015 (3)	0.020 (3)	0.018 (3)	0.003 (2)	0.006 (2)	0.003 (2)
C3A	0.026 (4)	0.037 (4)	0.016 (4)	0.013 (4)	0.009 (3)	0.007 (4)
C3B	0.026 (4)	0.037 (4)	0.016 (4)	0.013 (4)	0.009 (3)	0.007 (4)
C4	0.009 (3)	0.016 (3)	0.012 (3)	0.003 (2)	0.002 (2)	0.001 (2)
C5	0.008 (3)	0.016 (3)	0.016 (3)	0.001 (2)	0.004 (2)	0.003 (2)

### Geometric parameters (Å, °)

**Table:**

Gd—O1	2.575 (4)	O7—H7B	0.83 (7)
Gd—O1^i^	2.473 (4)	C1—C2	1.489 (8)
Gd—O2	2.569 (4)	C2—C3A	1.538 (10)
Gd—O2^ii^	2.430 (4)	C2—C3B	1.57 (3)
Gd—O3	2.403 (4)	C2—H2A	0.9700
Gd—O4^iii^	2.385 (4)	C2—H2B	0.9700
Gd—O5	2.374 (4)	C2—H2C	0.9700
Gd—O6^iv^	2.540 (4)	C2—H2D	0.9700
Gd—O7	2.420 (4)	C3A—C3A^v^	1.510 (16)
O1—C1	1.274 (7)	C3A—H3A1	0.9700
O2—C1	1.279 (6)	C3A—H3A2	0.9700
O3—C4	1.252 (7)	C3B—C3B^v^	1.54 (5)
O4—C4	1.249 (6)	C3B—H3B1	0.9700
O5—C5	1.253 (6)	C3B—H3B2	0.9700
O6—C5	1.246 (7)	C4—C4^iii^	1.558 (10)
O7—H7A	0.84 (6)	C5—C5^iv^	1.546 (10)
			
O5—Gd—O4^iii^	89.95 (13)	C4—O3—Gd	118.6 (3)
O5—Gd—O3	133.53 (13)	C4—O4—Gd^iii^	119.1 (3)
O4^iii^—Gd—O3	68.29 (13)	C5—O5—Gd	123.7 (3)
O5—Gd—O7	78.95 (14)	C5—O6—Gd^iv^	117.9 (3)
O4^iii^—Gd—O7	142.92 (13)	Gd—O7—H7A	122 (5)
O3—Gd—O7	141.60 (14)	Gd—O7—H7B	139 (5)
O5—Gd—O2^ii^	92.50 (13)	H7A—O7—H7B	97 (7)
O4^iii^—Gd—O2^ii^	142.01 (12)	O1—C1—O2	118.3 (5)
O3—Gd—O2^ii^	83.07 (12)	O1—C1—C2	120.4 (5)
O7—Gd—O2^ii^	74.35 (13)	O2—C1—C2	121.2 (5)
O5—Gd—O1^i^	81.91 (12)	O1—C1—Gd	59.4 (3)
O4^iii^—Gd—O1^i^	69.18 (13)	O2—C1—Gd	59.1 (3)
O3—Gd—O1^i^	122.76 (12)	C2—C1—Gd	174.0 (4)
O7—Gd—O1^i^	74.25 (14)	C1—C2—C3A	112.9 (5)
O2^ii^—Gd—O1^i^	148.60 (13)	C1—C2—C3B	112.6 (10)
O5—Gd—O6^iv^	65.44 (13)	C1—C2—H2A	109.0
O4^iii^—Gd—O6^iv^	70.90 (14)	C3A—C2—H2A	109.0
O3—Gd—O6^iv^	68.66 (13)	C3B—C2—H2A	135.3
O7—Gd—O6^iv^	131.77 (14)	C1—C2—H2B	109.0
O2^ii^—Gd—O6^iv^	75.79 (13)	C3A—C2—H2B	109.0
O1^i^—Gd—O6^iv^	127.57 (13)	C3B—C2—H2B	73.4
O5—Gd—O2	147.43 (13)	H2A—C2—H2B	107.8
O4^iii^—Gd—O2	122.50 (13)	C1—C2—H2C	109.1
O3—Gd—O2	69.27 (13)	C3A—C2—H2C	73.3
O7—Gd—O2	73.02 (13)	C3B—C2—H2C	109.1
O2^ii^—Gd—O2	64.38 (14)	H2B—C2—H2C	136.9
O1^i^—Gd—O2	105.56 (12)	C1—C2—H2D	109.1
O6^iv^—Gd—O2	124.28 (12)	C3A—C2—H2D	134.9
O5—Gd—O1	147.09 (13)	C3B—C2—H2D	109.1
O4^iii^—Gd—O1	80.01 (13)	H2C—C2—H2D	107.8
O3—Gd—O1	71.21 (13)	C3A^v^—C3A—C2	112.2 (8)
O7—Gd—O1	90.34 (13)	C3A^v^—C3A—H2C	148.3
O2^ii^—Gd—O1	114.63 (12)	C3A^v^—C3A—H3A1	109.2
O1^i^—Gd—O1	65.20 (14)	C2—C3A—H3A1	109.2
O6^iv^—Gd—O1	136.78 (12)	H2C—C3A—H3A1	91.7
O2—Gd—O1	50.45 (12)	C3A^v^—C3A—H3A2	109.2
O5—Gd—C1	159.72 (13)	C2—C3A—H3A2	109.2
O4^iii^—Gd—C1	100.86 (14)	H2C—C3A—H3A2	85.5
O3—Gd—C1	66.75 (14)	H3A1—C3A—H3A2	107.9
O7—Gd—C1	82.15 (15)	C3B^v^—C3B—C2	108 (2)
O2^ii^—Gd—C1	89.45 (14)	C3B^v^—C3B—H3B1	110.1
O1^i^—Gd—C1	85.94 (14)	C2—C3B—H3B1	110.1
O6^iv^—Gd—C1	134.31 (13)	C3B^v^—C3B—H3B2	110.1
O2—Gd—C1	25.31 (13)	C2—C3B—H3B2	110.1
O1—Gd—C1	25.21 (14)	H3B1—C3B—H3B2	108.4
C1—O1—Gd^i^	133.0 (3)	O4—C4—O3	126.3 (5)
C1—O1—Gd	95.4 (3)	O4—C4—C4^iii^	117.1 (6)
Gd^i^—O1—Gd	114.80 (14)	O3—C4—C4^iii^	116.6 (6)
C1—O2—Gd^ii^	147.7 (3)	O6—C5—O5	127.0 (5)
C1—O2—Gd	95.5 (3)	O6—C5—C5^iv^	116.6 (6)
Gd^ii^—O2—Gd	115.62 (14)	O5—C5—C5^iv^	116.4 (6)
			
O5—Gd—O1—C1	−140.5 (3)	O3—Gd—O5—C5	−8.0 (5)
O4^iii^—Gd—O1—C1	145.4 (3)	O7—Gd—O5—C5	148.3 (4)
O3—Gd—O1—C1	75.1 (3)	O2^ii^—Gd—O5—C5	74.8 (4)
O7—Gd—O1—C1	−70.5 (3)	O1^i^—Gd—O5—C5	−136.2 (4)
O2^ii^—Gd—O1—C1	2.4 (3)	O6^iv^—Gd—O5—C5	1.7 (4)
O1^i^—Gd—O1—C1	−143.0 (4)	O2—Gd—O5—C5	117.5 (4)
O6^iv^—Gd—O1—C1	97.7 (3)	O1—Gd—O5—C5	−138.6 (4)
O2—Gd—O1—C1	−3.1 (3)	C1—Gd—O5—C5	170.0 (4)
O5—Gd—O1—Gd^i^	2.5 (3)	Gd^i^—O1—C1—O2	−126.2 (4)
O4^iii^—Gd—O1—Gd^i^	−71.57 (16)	Gd—O1—C1—O2	5.5 (5)
O3—Gd—O1—Gd^i^	−141.88 (18)	Gd^i^—O1—C1—C2	55.3 (6)
O7—Gd—O1—Gd^i^	72.44 (17)	Gd—O1—C1—C2	−173.1 (4)
O2^ii^—Gd—O1—Gd^i^	145.34 (15)	Gd^i^—O1—C1—Gd	−131.6 (4)
O1^i^—Gd—O1—Gd^i^	−0.001 (2)	Gd^ii^—O2—C1—O1	−170.9 (4)
O6^iv^—Gd—O1—Gd^i^	−119.29 (18)	Gd—O2—C1—O1	−5.5 (5)
O2—Gd—O1—Gd^i^	139.9 (2)	Gd^ii^—O2—C1—C2	7.6 (9)
C1—Gd—O1—Gd^i^	143.0 (4)	Gd—O2—C1—C2	173.0 (4)
O5—Gd—O2—C1	139.9 (3)	Gd^ii^—O2—C1—Gd	−165.4 (6)
O4^iii^—Gd—O2—C1	−34.5 (3)	O5—Gd—C1—O1	86.4 (5)
O3—Gd—O2—C1	−79.2 (3)	O4^iii^—Gd—C1—O1	−34.7 (3)
O7—Gd—O2—C1	108.2 (3)	O3—Gd—C1—O1	−95.2 (3)
O2^ii^—Gd—O2—C1	−171.4 (4)	O7—Gd—C1—O1	107.9 (3)
O1^i^—Gd—O2—C1	40.5 (3)	O2^ii^—Gd—C1—O1	−177.9 (3)
O6^iv^—Gd—O2—C1	−122.4 (3)	O1^i^—Gd—C1—O1	33.2 (3)
O1—Gd—O2—C1	3.1 (3)	O6^iv^—Gd—C1—O1	−108.5 (3)
O5—Gd—O2—Gd^ii^	−48.6 (3)	O2—Gd—C1—O1	174.4 (5)
O4^iii^—Gd—O2—Gd^ii^	136.96 (15)	O5—Gd—C1—O2	−88.0 (5)
O3—Gd—O2—Gd^ii^	92.21 (16)	O4^iii^—Gd—C1—O2	150.9 (3)
O7—Gd—O2—Gd^ii^	−80.42 (17)	O3—Gd—C1—O2	90.4 (3)
O2^ii^—Gd—O2—Gd^ii^	0.0	O7—Gd—C1—O2	−66.5 (3)
O1^i^—Gd—O2—Gd^ii^	−148.11 (15)	O2^ii^—Gd—C1—O2	7.7 (3)
O6^iv^—Gd—O2—Gd^ii^	49.0 (2)	O1^i^—Gd—C1—O2	−141.2 (3)
O1—Gd—O2—Gd^ii^	174.5 (2)	O6^iv^—Gd—C1—O2	77.1 (3)
C1—Gd—O2—Gd^ii^	171.4 (4)	O1—Gd—C1—O2	−174.4 (5)
O5—Gd—O3—C4	−72.7 (4)	O1—C1—C2—C3A	66.3 (7)
O4^iii^—Gd—O3—C4	−5.0 (4)	Gd^iii^—O4—C4—O3	−175.4 (4)
O7—Gd—O3—C4	146.7 (3)	Gd^iii^—O4—C4—C4^iii^	5.3 (7)
O2^ii^—Gd—O3—C4	−159.5 (4)	Gd—O3—C4—O4	−175.0 (4)
O1^i^—Gd—O3—C4	39.7 (4)	Gd—O3—C4—C4^iii^	4.3 (7)
O6^iv^—Gd—O3—C4	−82.1 (4)	Gd^iv^—O6—C5—O5	178.4 (4)
O2—Gd—O3—C4	135.3 (4)	Gd^iv^—O6—C5—C5^iv^	−0.3 (7)
O1—Gd—O3—C4	81.5 (4)	Gd—O5—C5—O6	179.1 (4)
C1—Gd—O3—C4	108.1 (4)	Gd—O5—C5—C5^iv^	−2.1 (8)
O4^iii^—Gd—O5—C5	−67.3 (4)		

Symmetry codes: (i) −*x*, −*y*+1, −*z*+1; (ii) −*x*+1, −*y*+1, −*z*+1; (iii) −*x*, −*y*, −*z*+1; (iv) −*x*, −*y*, −*z*; (v) −*x*+1, −*y*+1, −*z*+2.

### Hydrogen-bond geometry (Å, °)

**Table:**

*D*—H···*A*	*D*—H	H···*A*	*D*···*A*	*D*—H···*A*
O7—H7A···O3^ii^	0.84 (6)	2.00 (5)	2.799 (4)	160
O7—H7B···O6^vi^	0.83 (7)	2.07 (7)	2.888 (5)	168

Symmetry codes: (ii) −*x*+1, −*y*+1, −*z*+1; (vi) −*x*, −*y*+1, −*z*.
